# Footprint analysis of CO_2_ in microbial community succession of raw milk and assessment of its quality

**DOI:** 10.3389/fnut.2023.1285653

**Published:** 2023-12-19

**Authors:** Anran Zheng, Jun Liu, Mengsong Wang, Ningxia Bu, Dunhua Liu, Chaokun Wei

**Affiliations:** ^1^School of Animal Science and Technology, Ningxia University, Yinchuan, China; ^2^School of Life Science, Hubei Normal University, Huangshi, China; ^3^School of Food Science and Engineering, Ningxia University, Yinchuan, China

**Keywords:** CO_2_ treatment, raw milk, microbial community succession, milk quality, flavor

## Abstract

With the growing production of raw milk, interest has been increasing in its quality control. CO_2_, as a cold processing additive, has been studied to extend the cold storage period and improve the quality of raw milk. However, it is yet uncertain how representative microbial species and biomarkers can succeed one another at distinct critical periods during refrigeration. Therefore, the effects of CO_2_ treatment on the succession footprint of the microbial community and changes in quality during the period of raw milk chilling were examined by 16S rRNA analysis combined with electronic nose, and electronic tongue techniques. The results indicated that, the refrigeration time was shown to be prolonged by CO_2_ in a concentration-dependent way. And CO_2_ treatment was linked to substantial variations in beta and alpha diversity as well as the relative abundances of various microbial taxa (*p* < 0.01). The dominant bacterial phylum Proteobacteria was replaced with Firmicutes, while the major bacterial genera *Acinetobacter* and *Pseudomonas* were replaced with lactic acid bacteria (LAB), including *Leuconostoc*, *Lactococcus*, and *Lactobacillus*. From the perspective of biomarkers enriched in CO_2_-treated sample, almost all of them belong to LAB, no introduction of harmful toxins has been found. The assessment of the quality of raw milk revealed that CO_2_ improved the quality of raw milk by lowering the acidity and the rate of protein and fat breakdown, and improved the flavor by reducing the generation of volatiles, and increasing umami, richness, milk flavor and sweetness, but reducing sourness. These findings offer a new theoretical foundation for the industrial use of CO_2_ in raw milk.

## 1 Introduction

Raw milk is rich in protein, fat, lactose and other nutrients and provides an excellent growth medium for microorganisms in milk (1). Therefore, raw milk must be refrigerated before processing to inhibit the propagation of microorganisms. During this period, the growth of most microorganisms is inhibited, but could not be terminated. With the passage of time, the propagated microorganisms act on the raw milk system, affecting its shelf life, nutritional value, nutritional quality, sensory flavor and health benefit, all of which are of particular concern to consumers (2). Especially *Pseudomonas* and *Acinetobacter*, which were considered to be the most abundant and harmful spoilage bacteria in raw milk (3), produce heat-resistant proteases and lipases that cannot be completely inactivated during heat treatment before processing, resulting in the decreased yield and quality of dairy products, such as high-temperature sterilized milk, cheese, yogurt and ice cream (4). As a result of the rise in cattle farming, longer storage times for milk during transportation and refrigeration have resulted from the consolidation of numerous small dairy manufacturing facilities into larger ones (5). In order to increase the storage time and quality of milk and dairy products generated from it, it is crucial to prevent microbial growth during raw milk refrigeration.

Compared to traditional thermal technology, non-thermal technology can suppress the growth of harmful microorganisms through non-heating treatment at ambient temperature (6), reducing the damage to food nutrition and sensory flavor caused by heat. Therefore, CO_2_, as a non-thermal antibacterial additive (7), has attracted researchers’ attention due to its natural origin (8), safety (9), simplicity and economy (10), as well as its easy removal from milk (8). It was reported that, adding CO_2_ in raw milk can prolong the cold storage period by inhibiting the lag, exponential and stationary growth phases of bacteria (11), especially gram-negative bacteria, such as psychrophilic bacteria (12), meanwhile the proteolysis and lipolysis were significantly reduced compared with untreated raw milk (13). Although the effects of CO_2_ on microorganisms in raw milk have been studied for decades, most of these have been traditional culture-dependent methods, leading to a significant underestimation of microbial diversity and CO_2_’s antibacterial effect, as the vast majority (approximately 99%) of microorganisms in nature cannot be cultured. With the development of gene sequencing technology unrelated to cultivation, a large number of unculturable microorganisms have been discovered, greatly improving people’s understanding of microbial communities (14). In recent years, researchers have further revealed the impact of CO_2_ on the microbial community structure of raw milk through denaturing gradient gel electrophoresis (DGGE) and next-generation amplification sequencing technology (14, 15). In addition, the effect of adding CO_2_ on milk powders, milk protein concentrate powders, yogurt and cheese has also been studied, which further confirmed that the effect of CO_2_ on the quality of dairy products was beneficial (16–21).

However, to our knowledge, little is known about how microbial communities evolve during cold storage and the dynamic succession of populations during storage of CO_2_-treated raw milk. This information may offer new insights into the time-specific features of microbial community succession. This knowledge is crucial because the types of microorganisms and their metabolites may have a direct impact on the sensory quality and shelf life of raw milk and dairy products. The present study used 16S rRNA gene sequencing technologies to identify the microbial communities’ succession footprint and other indicators during storage, and investigate how CO_2_ affects the quality of raw milk. The findings may improve our understanding of the variations in microbes between raw milk samples that has been chilled with CO_2_ and the control, as well as offer some theoretical guidance for how CO_2_ should be used in raw milk refrigeration.

## 2 Materials and methods

### 2.1 Raw milk collection and treatments

Fresh raw milk (the somatic cells <2 × 10^5^/mL, total bacteria count <10^3^ cfu/mL)was collected aseptically from milk tanks at Helan Mountain Dairy Farm, Ningxia, Yinchuan, China in June 2022, refrigerated at 4°C and transported to the laboratory within 1 h. Then, referring to the method of Ma et al. (13), a known mass of solid CO_2_ (dry ice, food grade) was added to 500 mL PET bottles (Servicebio, Wuhan, China) containing 300 mL of raw milk to achieve CO_2_ concentrations of 0, 500, 1,000 and 2,000 ppm, respectively. Each group of samples was divided into 100 mL aliquots in sterile sealed PET bottles and stored at 4°C. One aliquot was used for analysis each day. The day of sample collection was defined as day 0.

### 2.2 Bacterial growth studies

The total bacterial count (TBC) was measured by the spread plate method (22). Briefly, 1 mL sample was taken and serially diluted 10 times with 0.85% sterile saline. Then the 200 uL dilutions were spread onto Plate Count Agar (PCA) and cultured at 37°C for 48 h. Each sample was also spread onto MRS medium (cultured at 36°C for 72 h), eosin methylene blue (EMB) medium (cultured at 36°C for 24 h) and the psychrophilic count agar (cultured at 6.5°C for 10 days) to monitor the growth of LAB, *Escherichia coli* and *Psychrophilic bacteria*. Three parallel samples were taken for each sample.

Analysis of a sample was terminated when it was spoiled. Spoilage was defined by the TBC reaching a threshold of 6 lg (cfu/mL) (14).

### 2.3 Measurements of protein, fat and lactose content

The contents of fat, protein and lactose in raw milk were measured by using a rapid milk composition analyzer (Lactoscan SLP, Hangzhou, China). Briefly, after preheating the instrument for 10 min, distilled water and milk sample at 35–40°C were used to automatically clean the instrument. Then the sample was preheated at 25°C for 15 min before measurements according to the system program. Three parallel samples were taken for each sample.

### 2.4 Acidity measurement

CO_2_-treated samples were degassed using stirring to remove the effects of CO_2_ on the sample prior to measuring acidity (23). A raw milk sample (100 mL) was placed into a 250 mL beaker and stirred at a low speed with a magnetic stirrer until the pH value was no longer changed. Usually, the duration was less than 20 min. Three parallel samples were taken for each sample.

The milk acidity was determined according to the phenolphthalein indicator method in the Chinese National Food Safety Standard GB5009.239-2016 (24). Briefly, 10 g of milk sample were put into a 150 mL conical bottle, 20 mL of distilled water were added, boiled and cooled to room temperature and mixed well. Then 2 mL of phenolphthalein indicator were added, mixed well and titrated with 0.1 mol/L NaOH solution until the color of the sample became similar to that of the reference solution. The whole titration process was completed within 45 s. The titration volume of sodium hydroxide standard solution consumed was recorded. The titration volume of the consumed sodium hydroxide standard solution was recorded, and the acidity of the milk sample was calculated according to the following equation:

$$X=c\times \left(V_{1}:V_{0}\right)\times 100/m\times 0.1$$

Where *X* is the acidity of the sample (°T); *c* is the concentration of NaOH standard solution (mol/L); *V*_1_ is the volume of NaOH standard solution consumed during the titration of the sample (mL); *V*_0_ is the volume of NaOH standard solution consumed in the blank (mL); *m* is the mass of the sample (g).

### 2.5 Determination of flavor

#### 2.5.1 Volatile compounds analysis by electronic nose

Various volatile compounds were identified using a PEN3 Portable Electronic nose (E-nose) (Airsense Analytics GmbH, Schwerin, Germany) with 10 different MOS sensors to provide selectivity (Table 1). For the analysis, 10 mL of raw milk samples were put into a 30 mL headspace sample vial and equilibrated by incubation at room temperature (25 ± 2°C) for 30 min. Then, the E-nose probe was inserted to determine the volatile components in the milk. The relevant parameters of the electronic nose were as follows: sampling interval of 180 s, flushing time of 60 s, zero setting time of 10 s, detection time of 120 s, carrier gas flow rate of 300 mL/min, and injection flow rate of 300 mL/min. The E-nose sensor stabilized after 110 s, and 111, 112, and 113 s were selected as the information collection time. Five parallel samples were taken for each sample.

**Table 1:** Sensor properties of the E-nose sensor.

Sensor number	Sensor name	Descriptions
1	W1C	Sensitive to aromatic, benzene
2	W5S	Sensitive to nitrogen oxides
3	W3C	Sensitive to ammonia and aromatic compounds
4	W6S	Sensitive to hydrogen
5	W5C	Sensitive to alkanes, aromatic compounds, less polar compounds
6	W1S	Sensitive to methane and hydrocarbons
7	W1W	Sensitive to many terpenes and sulfides compound
8	W2S	Sensitive to alcohols, aldehydes and ketones
9	W2W	Sensitive to organic sulfides, aromatic compounds
10	W3S	Sensitive to long-chain alkanes

#### 2.5.2 Analysis of taste properties by electronic tongue

The taste properties in raw milk were assessed by SA-402B Electronic Tongue (E-tongue) (Insent Inc., Japan), comprising a sensor array, and data acquisition system and analysis system. The sensor array has six taste sensors, including C00, AAE, CA0, AE1, CT0, and GL1 for bitterness, umami, sourness, astringency, saltiness and sweetness, respectively, and two reference electrodes.

The CO_2_-treated samples were first degassed using stirring (23). Then 40 mL of raw milk were diluted with an equal volume of distilled water, followed by homogenization and filtration through a double layer of gauze. The resulting supernatant was collected, and subsequently, 70 mL of each sample were used for analysis. The test procedure was as follows: cleaning solution, 90 s, 120 s, 120 s; Conditioning solution, 30 s; and the detection time was set at 30 s. Three parallel samples were taken for each sample.

### 2.6 DNA extraction

Aliquots of samples (10 mL) were collected on the same day as plating, and stored at −80°C until DNA extraction with DNeasy PowerFood Microbial DNA Isolation Kit (QIAGEN, Dusseldorf, Germany). DNA extraction was performed according to the manufacturer’s instructions. The concentration and purity of the extracted DNA were determined by 1.2% agarose gel electrophoresis and nucleic acid purity tester (Liuyi, Beijing, China). Six parallel samples were taken for each sample.

### 2.7 PCR amplification and high throughput sequencing

PCR to amplify the 16S rRNA gene was performed with TransStart^®^ FastPfu DNA Polymerase (TransGen Biotech, Beijing, China) according to the manufacturer’s instructions. The variable V3-V4(a) region of the standard bacterial 16S rRNA gene as the target amplified region was amplified with the primers, ACTCCTACGGGAGGCAGCA and GGACTACHVGGGTWTCTAAT, and 2% agarose gel electrophoresis was used to verify the PCR products. Amplicons were extracted from the gels and purified using the VAHTSTM DNA Clean Beads (Vazyme, Nanjing, China). Concentrations of final products were determined with the Quant IT PicoGreen dsDNA Assay Kit (Invitrogen, Carlsbad, United States). High throughput sequencing was performed with the Illumina NovaSeq platform (Bioprofile Co., Ltd., Shanghai, China).

### 2.8 Bioinformatic analysis

Bioinformatics analysis was performed according to the procedure described by Koc et al. (25). Briefly, qiime cutadapt trim paired was used to cut the primer fragment of the sequence, and the sequence without matching primer discarded. Then DADA2 through qiime dada2 denoise paired was used for quality control, noise elimination and splicing. After obtaining the amplified sub sequence variant (ASV) table, the chimeric sequence was identified and removed. The feature sequence of each ASV was annotated into species by Naive Bayes classifier pre trained in QIIME2 software, through Silva database and classification skylearn algorithm. Alpha diversity and beta diversity were analyzed with QIIME2, and LEfSe analysis were performed with R software.

### 2.9 Statistical analysis

Results are expressed as mean ± standard deviation. Data were analyzed by two-way ANOVA using IBM SPSS 20.0. Significance was evaluated at *p < 0.05 or **p < 0.01. Images were processed and generated using Origin 2023 software or R software.

## 3 Results

### 3.1 CO_2_ decreased the total bacterial count in raw milk

**Figure 1:**
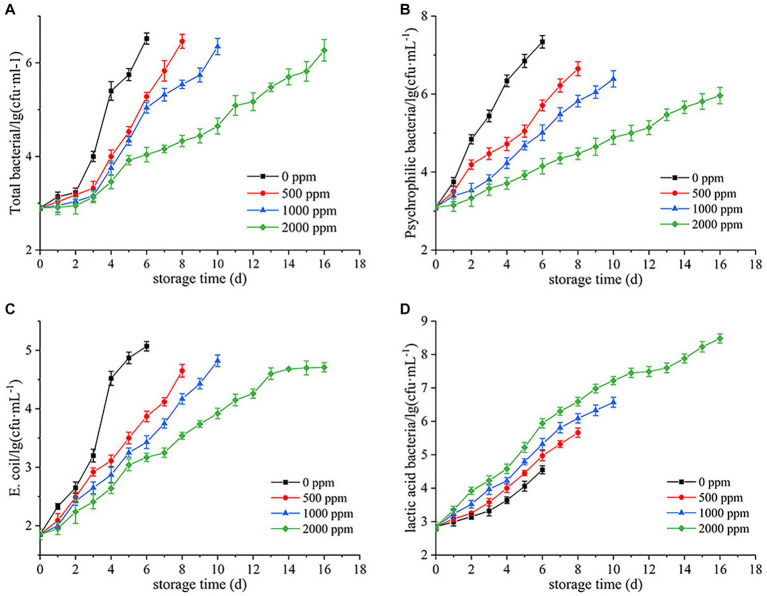
Inhibition of bacterial growth by CO_2_ in raw milk stored at 4°C. **(A)** Total bacterial count. **(B)** Psychrophilic bacteria count. **(C)** Escherichia coli count. **(D)** Lactic acid bacteria count.

As shown in Figure 1, the TBC in all groups increased with refrigeration time, but the CO_2_-treated groups showed significantly lower levels than the untreated group (0 ppm), especially after 3 d (Figure 1A). The untreated group spoiled at day 6, with a TBC of 6.52 lg (cfu/mL). Meanwhile, the 500 and 1,000 ppm groups were 5.28 and 5.04 lg (cfu/mL), respectively, while the 2000 ppm group only had 4.04 lg (cfu/mL), which was close to the untreated group [4.00 lg (cfu/mL)] at day 3. With the increase of refrigeration time, the 500, 1,000 and 2,000 ppm treated groups spoiled at day 8, 10, and 16, with TBC of 6.46, 6.35, and 6.24 lg (cfu/mL), respectively. The result showed that dissolving CO_2_ in raw milk inhibited the growth of TBC, and prolonged the cold storage period of raw milk with increasing concentrations of CO_2_. Moreover, CO_2_ exhibited a good inhibitory effect on psychrophilic bacteria and *Escherichia coli* (Figures 1B,C). Interestingly, LAB grew faster at high concentrations of CO_2_ (Figure 1D).

### 3.2 CO_2_ affected microbial community diversity of raw milk

**Figure 2:**
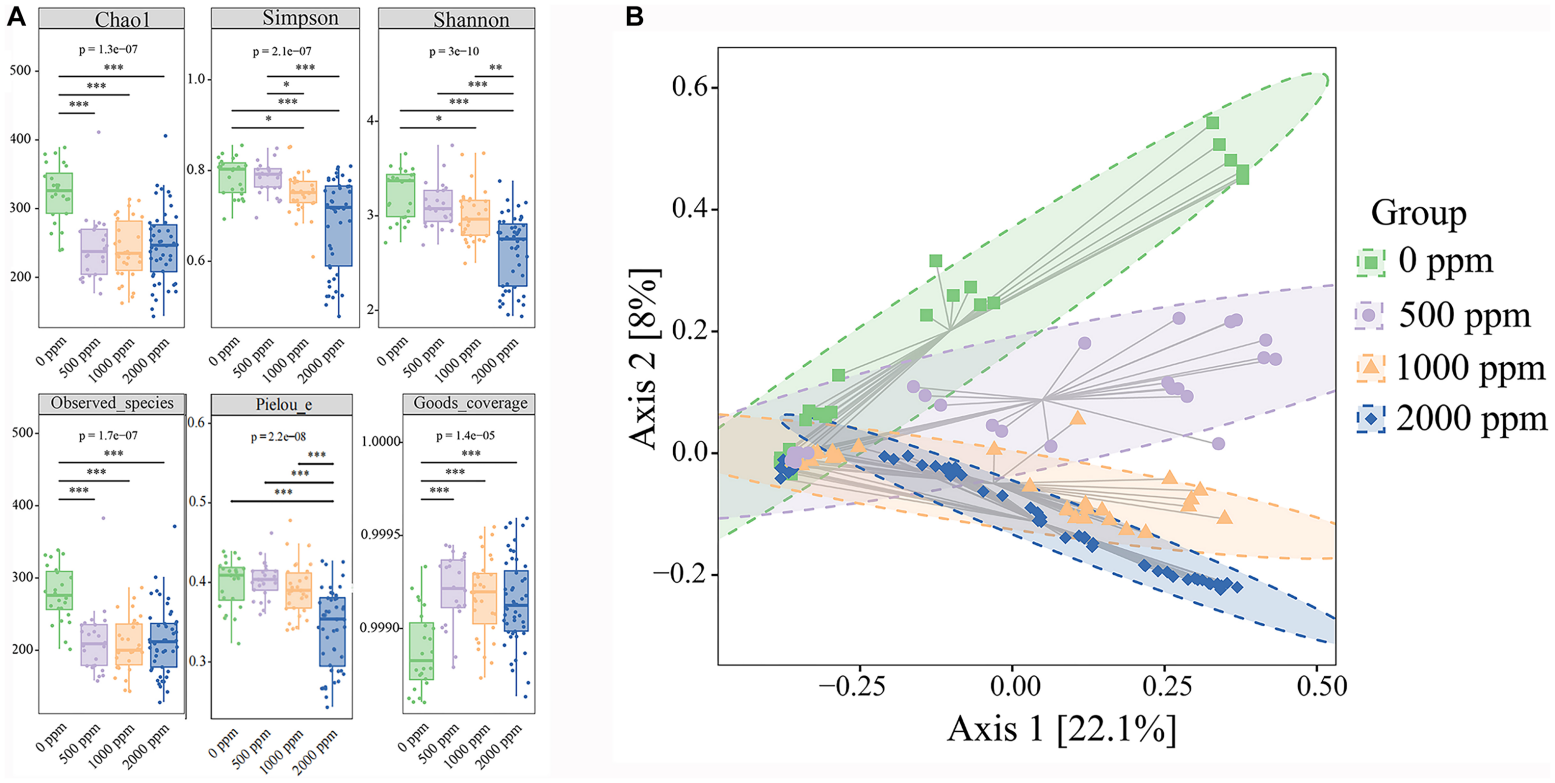
Effect of CO_2_ concentration on microbial community diversity of raw milk. **(A)** Alpha diversities. Kruskal-Wallis and dunn’ test were used to verify the significance of the difference. **(B)** Beta Diversity. Principal Coordinate Analysis (PCoA) using Bray–Curtis dissimilarity shows separation between groups. Eclipses indicate 95% confidence intervals around samples from each group. Significant differences were recorded by*p < 0.05,**p < 0.01, and ***p < 0.001.

**Figure 3:**
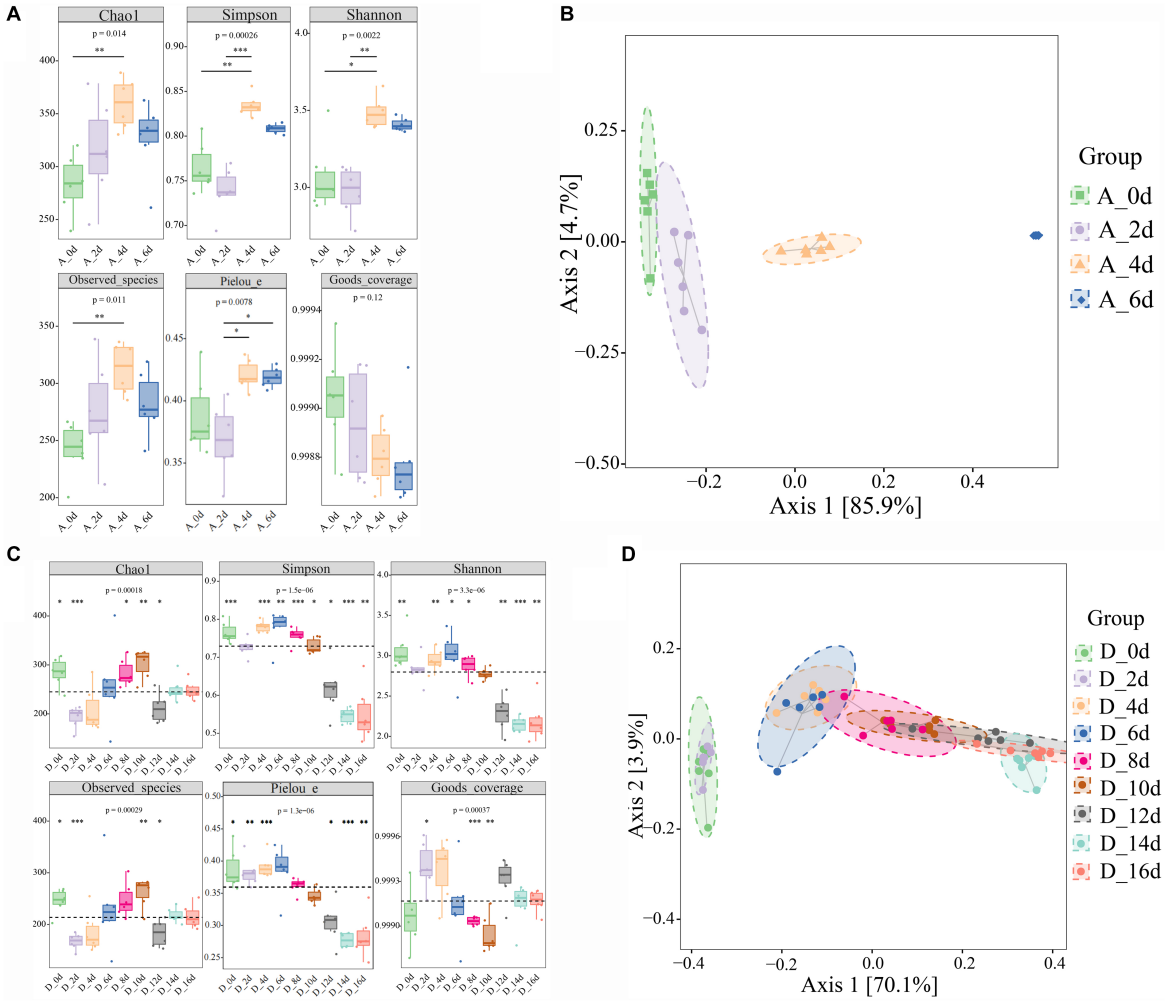
Effect of CO_2_ treatment on microbial community diversity of raw milk. **(A,B)** Alpha diversities. Kruskal-Wallis and dunn’ test was used to verify the significance of the difference. **(C,D)** Beta Diversity. Principal Coordinate Analysis (PCoA) using Bray–Curtis dissimilarity showed separation between groups. Eclipses indicate 95% confidence intervals around samples from each group. A_0d, sample of 0 ppm at 0 d, D_0d, sample of 2,000 ppm at 2 d, and so on. Significant differences were recorded by *p < 0.05, **p < 0.01, and ***p < 0.001.

A total of approximately 10,489,975 raw reads were obtained from 126 samples. After the filtering process, 8,912,557 valid reads with an average length of 429 bp were retained. By removing 2 ASVs corresponding to unclassified sequences, a total of 8,364 ASVs were obtained, including 1 domain (bacteria), 28 phyla, 87 classes, 173 orders, 315 families and 657 genera. As shown in Figure 2A, the Goods coverage were all close to 1 (>0.99), indicating that most microorganisms were observed in the samples and the sequencing results were accurate. Compared with the untreated group, all alpha diversity indices in CO_2_-treated group tended to decrease. The result showed that CO_2_ reduced the diversity, richness and Pielou’s evenness of samples significantly (*p* < 0.05, *p* < 0.01, and *p* < 0.001, respectively). During the cold storage (Figure 3A), for the untreated group, Chao1, Observed species, Shannon, Simpson and Pielou’s evenness all reached the maximum values at day 4, and then decreased slowly at day 6. In contrast, as shown in Figure 3C, the Chao1, Observed species, Shannon and Simpson in the raw milk treated with 2,000 ppm CO_2_ were significantly lower at day 2 (*p* < 0.01), and then gradually increased with the storage time. Chao1 and Observed species reached the maximum at day 10, and Shannon and Simpson reached the maximum at day 6, and then tended to decrease. Pielou’s evenness increased from day 0 to day 6. These results showed that the diversity, richness and evenness of the microbial community in the raw milk were changed with the storage time, and CO_2_ treatment delayed the arrival of the peak values.

In the raw milks treated with CO_2_ at various doses, variations in the community composition were examined using principal coordinate analysis (PCoA). Using permutational multivariate analysis of *variance* (PERMANOVA), the community composition in raw milks treated with CO_2_ at various levels significantly distinguished the treated raw milk from the untreated raw milk (Figure 2B). In the untreated group and the 2,000 ppm group, there appeared to be a divergence in the community composition over the course of the cold storage (Figures 3B,D).

### 3.3 CO_2_ changed the microbial community structure of raw milk

#### 3.3.1 The effect of CO_2_ concentration on the microbial community structure

**Figure 4:**
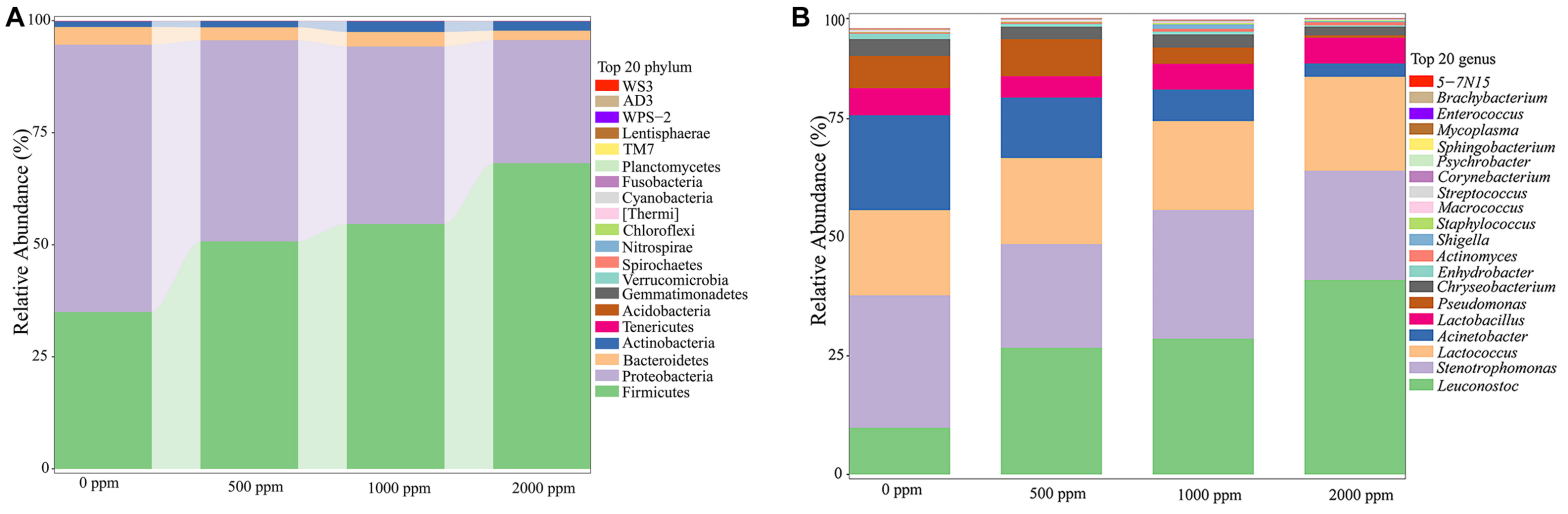
Analysis of bacterial community profiling of raw milks treated with various concentrations of CO_2_. **(A)** Bacterial communities at the phyla level (Top 20). **(B)** Bacterial communities at the genus level (Top 20).

As shown in Figure 4A, the most dominant phyla were Firmicutes and Proteobacteria (94.24–95.27%), followed by Bacteroidetes and Actinobacteria in each group. As shown in Supplementary Table S1, compared to the untreated group, the relative abundance of Firmicutes in CO_2_-treated groups increased significantly (*p* < 0.01), and the relative abundance of Proteobacteria decreased significantly (*p* < 0.01) in a dose-dependent manner. The relative abundance of Bacteroidetes in CO_2_-treated groups except the 1,000 ppm group was significantly reduced (*p* < 0.05 or *p* < 0.01), and the relative abundance of Actinobacteria except the 500 ppm was significantly increased (*p* < 0.01) when compared to the untreated group.

The dominant bacterial communities at genus level were *Leuconostoc*, *Stenotrophomonas*, *Lactococus*, *Acinetobacter*, *Lactobacillus*, *Pseudomonas*, et al. (Figure 4B). A number of significant differences were observed (Supplementary Table S2). The *Leuconostoc*, *Actinomyces and Lactococus* in CO_2_-treated groups increased significantly (*p* < 0.01 or *p* < 0.05), while the *Acinetobacter*, *Pseudomonas*, *Chryseobacterium* and *Enhydrobacter* decreased significantly compared to the untreated group (*p* < 0.05 or *p* < 0.01).

#### 3.3.2 The footprint analysis of microbial communities over time

**Figure 5:**
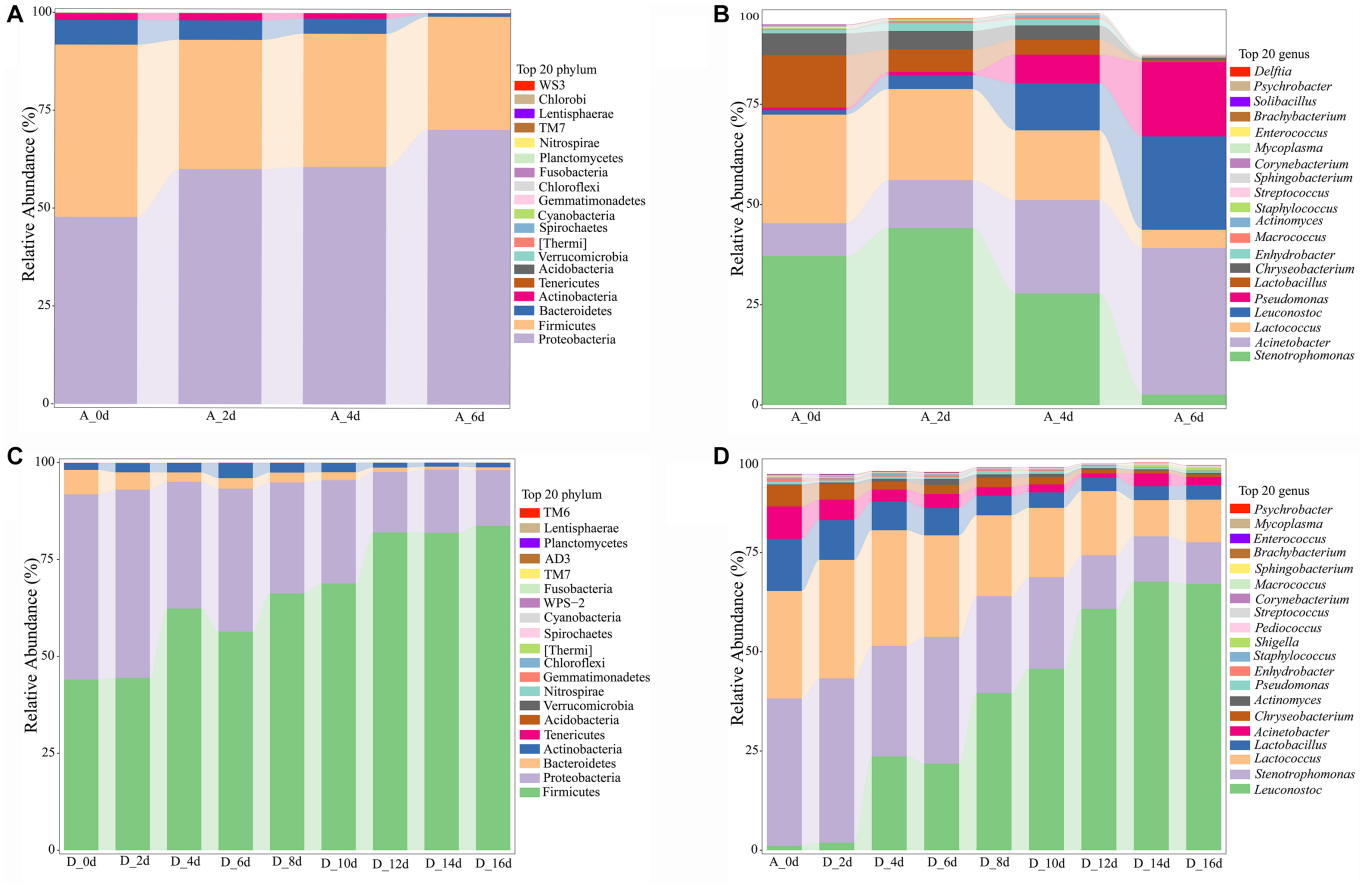
Analysis of bacterial community profiling of raw milk with time during cold storage. **(A)** Bacterial communities at the phyla level of the untreated group (0 ppm). **(B)** Bacterial communities at the genus level of untreated group. **(C)** Bacterial communities at the phyla level of CO_2_ treated raw milk (2,000 ppm). **(D)** Bacterial communities at the genus level of CO_2_ treated raw milk. A_0d, sample of 0 ppm at 0 d; D_0d, sample of 2,000 ppm at 0 d, and so on.

The footprint of microbial communities succession in the untreated (0 ppm) and treated (2,000 ppm) raw milk during the whole refrigerated period was shown in Figure 5. It was found that there were 4 phylum with abundance greater than 1% in both groups (Figures 5A,C). Firmicutes and Proteobacteria were the most dominant phylum, followed by Bacteroides and Actinobacteria. The difference was that the abundance of Proteobacteria in the untreated sample gradually increased with the storage time, until it reached 70.18% when it was spoiled. However, the abundance of Firmicutes in the treated sample increased gradually with the storage time, and reached 83.70% until spoiled. The results indicated that there was a growth competition between the two phylum.

**Figure 6:**
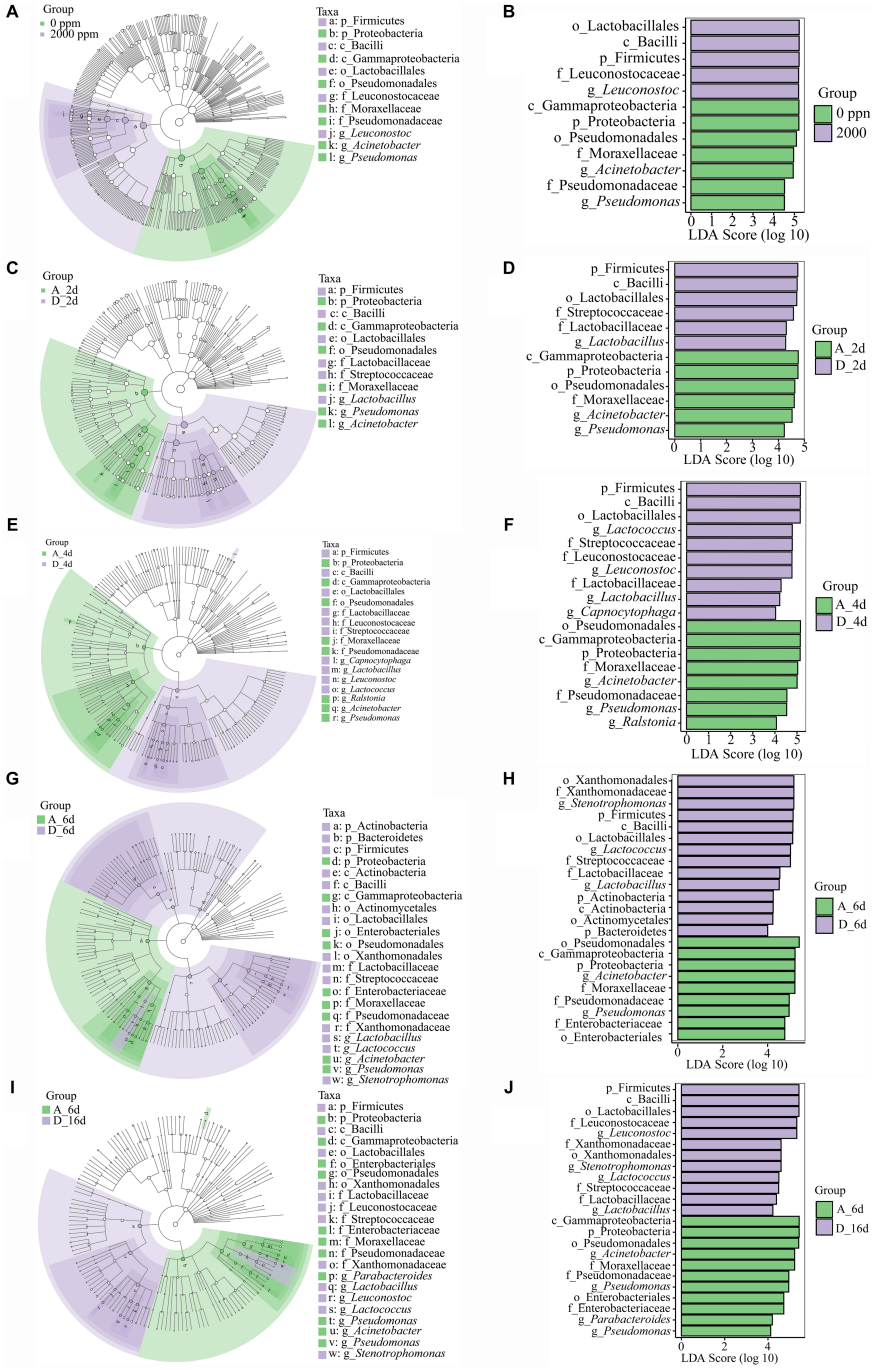
Linear discriminant analysis effect size (LEfSe) analysis to detect the biomarkers between the untreated raw milk (0 ppm) and CO_2_-treated raw milk (2,000 ppm). **(A–E)** Cladogram showing biomarkers of the significant and biological differences from the phylum level to the species level. **(F–J)** LDA scores of the biomarkers. A_2d, sample of untreated at day 2; D_2d, sample of CO_2_ treated at day 2, and so on.

At genus level (Figures 5B,D), there were 8 genera with abundance greater than 1%. For the untreated sample, the initial dominant bacteria were *Stenotrophomonas* (37.13%), *Lactococcus* (27.10%), and *Lactobacillus* (13.09%), followed by *Acinetobacter* (8.20%) and *Chryseobacterium* (5.40%). The relative abundance of *Lactobacillus* decreased to 5.61%, while *Stenotrophomona*s (44.10%), *Lactococcus* (22.74%), and *Acinetobacter* (11.98%) became the dominant genera after 2 days. At day 4, *Leuconostoc* and *Pseudomonas* increased from 1.1 and 0.68% to 11.70 and 7.15%, respectively, compared with day 0. However, the dominant bacteria were still *Stenotrophomonas* (27.80%), *Acinetobacter* (23.29%), and *Lactococcus* (17.43%), and the flora distribution was relatively uniform. The abundance of *Acinetobacter*, *Leuconostoc* and *Pseudomona*s increased rapidly to 36.56, 23.29, and 18.55% respectively, which became the dominant bacteria at day 6, when the raw milk was spoiled. By comparison, the *Leuconostoc* replaced *Stenotrophomonas* and *Lactococcus* as the most dominant bacteria with the storage time in the CO_2_-treated raw milk (Figure 6D).

#### 3.3.3 The dynamic impact of CO_2_ on microbial communities

In order to monitor the dynamic impact of CO_2_ treatment on raw milk during refrigeration, the CO_2_-treated (2,000 ppm) sample was compared to the untreated (0 ppm) sample at day 2, 4 and 6, respectively (Supplementary Table S3). At day 2, the main genera of bacteria were *Stenotrophomonas, Lactococcus, Acinetobacter, Lactobacillus, Chryseobacterium*, and *Leuconostoc* in both groups. The relative abundances of *Lactobacillus* and *Lactococcus* were significantly higher in the CO_2_-treated sample than that in the untreated sample (*p* < 0.01 or *p* < 0.05), while a lower relative abundance of *Acinetobacter* and *Leuconostoc* was observed in the CO_2_-treated group (*p* < 0.01). At day 4, the relative abundance of 7 genera in the CO_2_-treated group changed greatly. *Leuconostoc, Lactococcus* and *Lactobacillus* were significantly higher (*p* < 0.01), while *Acinetobacter*, *Pseudomonas*, *Enhydroactor*, and *Chryseobacterium* were significantly lower when compared with the untreated group (*p* < 0.01). At day 6, the relative abundance of 6 genera in the CO_2_-treated group changed greatly as compared with the untreated group. CO_2_ treatment significantly lowered the relative abundance of *Acinetobacter* and *Pseudomonas* (decreased by 10.33-fold and 44.17-fold, respectively, *p* < 0.01), while the relative abundance of *Stenotrophomonas*, *Lactococcus*, *Lactobacillu*s, and *Chryseobacterium* was significantly higher (increased by 12.48-fold, 5.86-fold, 17.64-fold, and 3.35-fold, respectively, *p* < 0.01).

During the subsequent cold storage period, the relative abundance of *Leuconostoc* continued to increase, while the relative abundance of *Stenotrophomonas*, *Lactococcus*, *Chryseobacterium*, and *Actinomyces* continued to decrease, meantime the relative abundance of *Lactobacillus* and *Pseudomonas* also decreased slowly. Finally, CO_2_ picked different spoilage microorganisms in raw milk samples. Compared with the untreated group, the relative abundance of *Leuconostoc* in the CO_2_-treated group increased significantly to 67.12% (*p* < 0.01, Supplementary Table S3), which became the most dominant spoilage bacterium. Meantime the relative abundance of *Lactococcus* and *Stenotrophomonas* increased significantly to 10.73 and 10.48%, becoming the second dominant spoilage bacteria. Although the relative abundance of *Lactobacillus* in the CO_2_-treated group was 3.67%, it was only 0.39% in the untreated group. The relative abundance of *Acinetobacter* and *Pseudomonas* which were dominant spoilage bacteria in the untreated group decreased rapidly to 2.06 and 0.33%, respectively, (*p* < 0.01) in the CO_2_-treated group.

### 3.4 Species difference footprint analysis and markers

To better understand the dominance of specific bacteria in the CO_2_-treated group and untreated group during cold storage, the untreated (0 ppm) and CO_2_-treated (2,000 ppm) group were analyzed by Lefse analysis (LDA score > 4) (Figure 6). On the whole, *Leuconostoc*, Leuconostocaceae, Lactobacillales, Bacilli and Firmicutes significantly enriched in the CO_2_-treated group compared to the untreated group (Figures 6A,B). At day 2 (Figures 6C,D), there were notable distinguishing bacteria in various levels with Firmicutes, Bacilli, Lactobacillales, Streptococcaceae, Lactobacillaceae, and *Lactobacillus* in the CO_2_-treated group compared to the untreated group. At day 4 (Figures 6E,F), there were a total of 18 different bacteria at different levels, 9 of which were Firmicutes, Bacilli, Lactobacillales, Leuconostocaceae, Streptococcaceae, Lactobacillaceae, *Leuconostoc*, *Lactococcus* and *Lactobacillus* in the CO_2_-treated group. At day 6 (Figures 6G,H), the difference between the two groups became greater, with 23 notable distinguishing bacteria. Among them, *Stenotrophomonas*, *Lactococcus* and *Lactobacillus* became the biomarkers at genus level in the CO_2_-treated group.

It was found that CO_2_ significantly changed the spoilage bacteria of raw milk (Figures 6I,J). At phylum level, Proteobacteria was significantly enriched in the untreated group, while Firmicutes was significantly enriched in the CO_2_-treated group. Meanwhile, *Acinetobacter* and *Pseudomonas* were the spoilage biomarkers in the untreated group and *Leuconostoc*, *Stenotrophomonas*, *Lactococcus*, and *Lactobacillus* were the spoilage biomarkers in the CO_2_-treated group at genus level.

### 3.5 Contents of protein, fat and lactose, and acidity of raw milk

When the refrigeration time increased in the two groups, the contents of protein, fat and lactose all gradually dropped (*p* < 0.05, Table 2), and the acidity gradually increased (*p* < 0.05). Interestingly, in the CO_2_-treated sample, the rate of protein and fat oxidation was slower. Additionally, starting on day 4, the protein content differed significantly (*p* < 0.01) from the untreated group. Similar to this, the rate of acidity increase was slower and highly significant from 6 days compared to the untreated group in the CO_2_-treated group. In the CO_2_-treated group, lactose catabolism increased at a faster pace, and starting on day 4 (*p* < 0.01), the lactose content was significantly lower than that of the untreated group.

**Table 2:** Major physicochemical qualities in the control and CO_2_-treated raw milk.

	Protein (g/100 g)		Fat (g/100 g)		Lactose (g/100 g)		Acidity (°T)	
	0 ppm	2,000 ppm	0 ppm	2,000 ppm	0 ppm	2,000 ppm	0 ppm	2,000 ppm
0 d	3.63 ± 0.061^a^	3.63 ± 0.061^a^	3.98 ± 0.066^a^	3.98 ± 0.066^a^	4.95 ± 0.021^a^	4.95 ± 0.021^a^	16.00 ± 0.000^a^	16.00 ± 0.000^a^
2 d	3.56 ± 0.032^a^	3.61 ± 0.006^b^	3.81 ± 0.057^ab^	3.94 ± 0.059^a^	4.92 ± 0.026^a^	4.90 ± 0.025^a^	16.5 ± 0.000^ab^	16.33 ± 0.289^ab^
4 d	3.44 ± 0.061^b^	3.54 ± 0.046^bc^**	3.57 ± 0.015^b^	3.91 ± 0.02^ab^	4.83 ± 0.021^b^	4.7 ± 0.067^b^**	16.83 ± 0.289^b^	16.58 ± 0.382^bc^
6 d	3.22 ± 0.012^c^	3.49 ± 0.045^c^**	3.50 ± 0.023^b^	3.81 ± 0.532^ab^	4.78 ± 0.036^b^	4.67 ± 0.02^b^**	18 ± 0.000^c^	16.67 ± 0.289^bc^**
16 d	/	3.19 ± 0.015^d^	/	3.49 ± 0.04^b^	/	4.23 ± 0.044^b^	/	18.67 ± 0.289^d^

Different lowercase superscripts indicate significant differences between samples within the same treatment group (*p* < 0.05). * indicates a significant difference (*p* < 0.05) and ** indicates a highly significant difference (*p* < 0.01) between CO_2_ treated and control samples within the same treatment time groups.

### 3.6 Correlation between major microorganisms and physicochemical properties

**Figure 7:**
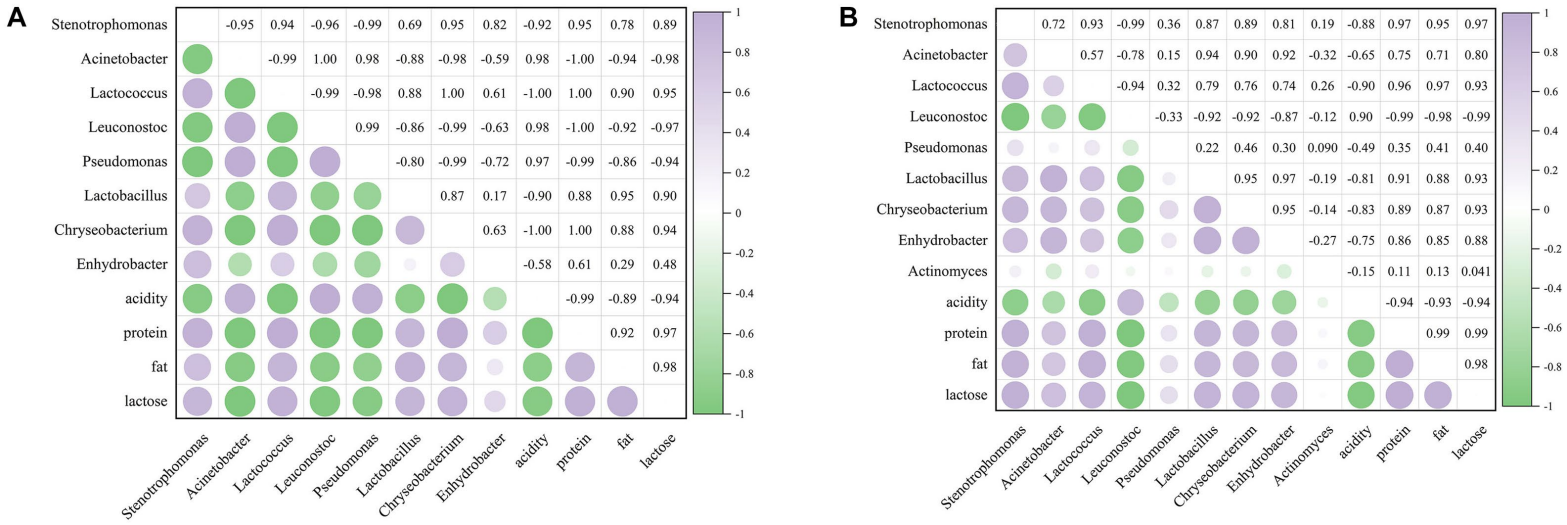
Correlation analysis between main bacterial genera and major physicochemical qualities. **(A)** The untreated sample. **(B)** The CO_2_ treated sample. The numbers in the heat map indicate the correlation index r, −1 < r < 1. | r | > 0.8 was considered as highly correlated, 0.5 < | r | < 0.8 as moderately correlated, 0.3 < | r | < 0.5 as poorly correlated, and | r | < 0.3 as not correlated.

To investigate the influence of microorganisms on raw milk quality during refrigeration, Pearson correlation analysis was performed between the main microorganisms (relative abundance >0.01) and physicochemical properties of raw milk during refrigeration. For the untreated group, the abundance of *Acinetobacter*, *Leuconostoc* and *Pseudomonas* was negatively correlated with the contents of protein, fat and lactose, but positively correlated with acidity (Figure 7A). *Pseudomonas* spp. was reported to be the main proteolytic strain at 7°C and 20°C (26). The correlation between *Enhydrobacter* and milk quality was weaker. For the CO_2_-treated group, as shown in Figure 7B, *Leuconostoc* mainly contributed to acidity; *Stenotrophomonas*, *Lactococcus*, *Lactobacillus*, *Chryseobacterium*, and *Enhydrobacter* had a strong correlation with the contents of protein, fat and lactose, followed by *Acinetobacter*.

### 3.7 CO_2_ improved the flavor of raw milk

#### 3.7.1 Volatile odors

**Figure 8:**
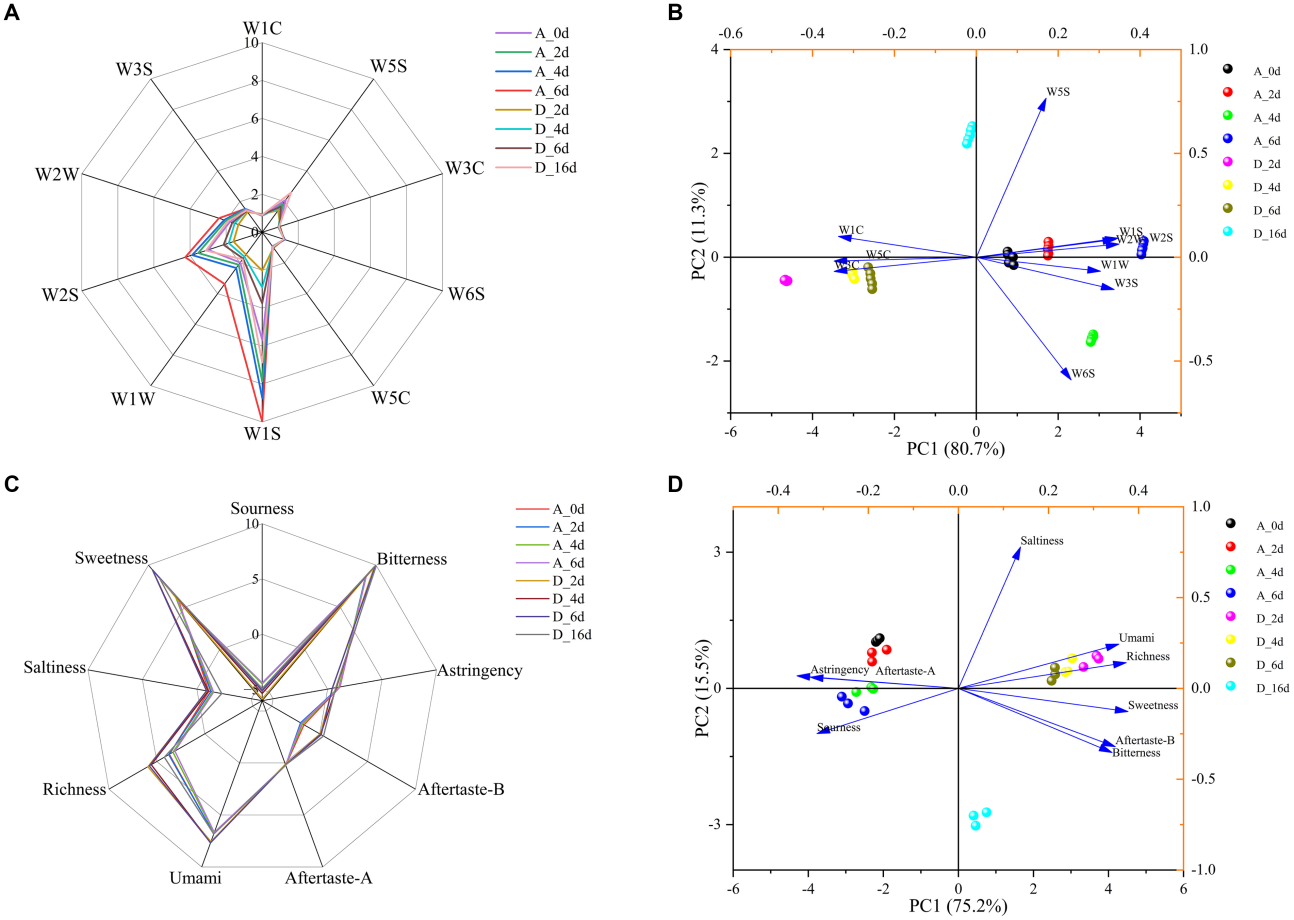
The effect of CO_2_ treatment on the flavor of raw milk. **(A)** Radar chart of volatile components in raw milk during refrigeration based on electronic nose. **(B)** PCA of volatile components in raw milk. **(C)** Radar chart of taste properties in raw milk during refrigeration based on electronic tongue. **(D)** PCA of taste properties in raw milk.

As shown in Figure 8A, there were significant differences in the odor profile curves of raw milk between the control group and CO_2_-treated group at each critical time point. The response values of W3S, W1C, W3C, W6S, W5C, and W5S sensors in the control group/CO_2_-treated group were not significantly different, indicating that some volatile compounds (alkanes, aromatic compounds, ammonia compounds, hydrogen, olefins and nitrogen oxides) were not affected by CO_2_ treatment or refrigeration time. The response values of W2W, W2S, W1W, and W1S sensors showed significant differences between samples, indicating that volatile compounds (aromatic compounds and organic sulfides, alcohols, aldehydes, ketones, hydrogen sulfide, methane and hydrocarbons) underwent significant changes with CO_2_ treatment or refrigeration time. Specifically, the contents of these compounds increased with the refrigeration time. In the CO_2_-treated group, the change patterns of these volatiles were similar to that of the control group, but the overall content was significantly lower than that of the control group.

PCA was used to analyze the E-nose data. As shown in Figure 8B, the control and CO_2_-treated samples were easily separated on both sides of the vertical axis. PC1 represented 80.7% of the total variance, while PC2 represented 11.3% of the total variance. The cumulative contribution rate of the two principal components was greater than 90%, which covered the vast majority of odor information in the samples. Obviously, W3C and W5C were associated with CO_2_-treated samples, while W2W, W2S, W1W, W1S, W3S, and W6S were associated with the control samples.

#### 3.7.2 Taste properties

For the control group, there was no significant difference in the taste profile curves at each key time point, accompanied by a decrease in richness, saltiness and freshness, with an increase in acidity (Figure 8C). For the CO_2_-treated samples, the trend of each taste profile was similar to that of the control samples over time. The differences were that the umami, richness, bitterness, after taste-B and sweetness were significantly greater than those of the control group, while the sourness was significantly lower. It is worth noting that, according to the manual of the E-tongue instrument, the “C00 Bitterness Sensor” was used to evaluate the “milk taste” of milk, reflecting the richness of the milk flavor.

PCA was used to analyze the E-tongue data. As shown in Figure 8D, the control and CO_2_-treated samples were easily separated on both sides of the vertical axis. PC1 represented 75.2% of the total variance, while PC2 represented 15.5% of the total variance. The cumulative contribution rate of the two principal components was greater than 90%, which covered the vast majority of taste information in the samples. Obviously, CO_2_-treated samples were associated with umami, richness, sweetness, bitterness (milk flavor) and aftertaste-B, while the control samples were associated with astringency, aftertaste-A and sourness.

## 4 Discussion

The finding that CO_2_ decreased microorganism development in raw milk in a concentration-dependent manner was in line with an earlier research (12). A similar finding was reported previously (27) in which raw chicken was maintained in a CO_2_ modified package. According to Devlieghere et al. (28) and Mélanie et al. (29), the amount of dissolved CO_2_ in the food matrix had a direct correlation with the bacteriostatic effect of CO_2_. However, the antibacterial mechanism of dissolved CO_2_ is still unclear. There were four primary hypotheses regarding bacterial inhibition (10): (1) The solubility of CO_2_ in lipids may disrupt bacterial membrane permeability. (2) Hydration reactions of CO_2_ may lead to a decrease in pH, causing both intracellular and environmental stress. (3) CO_2_, being a metabolite in numerous biochemical pathways, can result in wasteful expenditure of cellular energy. (4) CO_2_ has the potential to induce physiochemical alterations and regulate enzymes. Consequently, it is imperative to conduct further investigations into the specific mechanisms.

Analysis of alpha diversity showed that CO_2_ treatment reduced Chao1, Observed species and Shannon and Simpson index (Figure 2A), suggesting that CO_2_ treatment could lower the richness and diversity of microbial communities. This may be caused by the inhibition of microorganisms by carbonation (10). For the untreated sample, the richness and diversity of microorganisms reached the maximum at day 4, and then decreased at day 6. This may be because during the early stages of refrigeration, the raw milk provides sufficient nutrients for microorganisms to reproduce in large quantities (1), with the increase of refrigeration time, the trophic cells of thermophilic bacteria were deactivated and their growth was inhibited, while psychrophilic bacteria survived by adapting to low-temperature environments (30). When CO_2_ was added to the raw milk, the richness and diversity of microorganisms underwent complex changes (Figure 3B). Martin et al. (11) proposed a species-specific reaction to CO_2_ in raw milk. The impact of CO_2_ on microorganisms varies in different growth stages, including lag, log and stationary phases (10, 11). Therefore, it is speculated that the rapid change in the milk environment at the initial stage of CO_2_ addition may cause some microorganisms to become dormant because they could not adapt to the new environment, so the microbial richness and diversity decreased sharply on day 2. With the gradual adaptation of microorganisms to the milk environment, the microbial growth entered the log phase, so their richness and diversity tended to increase. With the increase of refrigeration time, some microorganisms entered a resting or even declining period, and the selective effect of CO_2_ on microorganisms (such as LAB) gradually increased, leading to a convergence of microorganisms in the dairy environment, and a decrease in diversity and richness. The β-diversity analysis showed that the samples treated with different concentrations of CO_2_ were significantly separated from each other, indicating that CO_2_ treatment can change the direction of microbial succession in raw milk. It is noteworthy that the samples in the control group were significantly separated at the critical time points, whereas the samples in the treated group were only significantly separated between day 2 and day 4, and the other samples at adjacent critical time points overlapped in the direction of succession, indicating that the addition of CO_2_ may make the microbes to change their succession more slowly between the groups, which was related to the selective inhibitory effect of CO_2_ on the microbes.

CO_2_ treatment led to a significant increase in the relative abundance of Firmicutes in raw milk, which is not surprising as this phylum is a large group of Gram-positive bacteria, mainly dominated by LAB (31, 32), including *Leuconostoc*, *Lactococcus* and *Lactobacillus*. It was observed that they can be selected by CO_2_ in raw milk, of which *Leuconostoc* was the most significant, followed by *Lactococcus*. This result was consistent with a previous study (14), showing that the selective effect of CO_2_ on microorganisms was related to the initial microbial community structure of raw milk. It’s known that LAB are suitable for growing under acidic conditions (33), CO_2_ was highly soluble in the liquid phase and caused a reduction in pH (7), which had a promoting effect on them. Moreover, a large number of Gram-negative bacteria in the sample were inhibited by CO_2_ (12), which weakened the inter-bacterial competition of LAB, thus providing better living conditions for LAB and promoting their growth and reproduction. Besides, a recent study reported that *Lactobacillus* and *Leuconostoc* could ferment sugars into acids and produce antibacterial substances that inhibit the growth of competing bacteria (*Acinetobacter* and *Pseudomonas*) (34). Another study also showed that LAB had a high tolerance to CO_2_ and can be selected from meat products packaged with CO_2_ modified atmosphere (35). Not surprisingly, the results of species difference footprint analysis showed that almost all biomarkers enriched in the CO_2_-treated milk were Firmicutes and LAB, which once again confirmed the selective effect of CO_2_ on Firmicutes and LAB. LAB, including *Streptococcus*, *Leuconostoc*, *Lactococcus*, and *Lactobacillus*, have been used in vegetables, meat and dairy products since the last century (36) and were widely recognized as safe (9). It is worth mentioning that the proportion of *Streptococcus* and *Staphylococcus*, which belong to Firmicutes, had an extremely small proportion in raw milk (< 0.13%), and the addition of CO_2_ resulted in a lower proportion of *Streptococcus* (<0.09%), while the proportion of *Staphylococcus* slightly increased (<0.3%). Actinobacteria is also a kind of Gram-positive bacteria, with *Actinomyces* being one of the main genera. It was found that CO_2_ significantly increased the proportion of Actinobacteria and *Actinomyces* in raw milk in our study. To our knowledge, few studies have reported the selective effect of CO_2_ on *Actinomyces*.

Proteobacteria is the largest phylum of bacteria, belonging to Gram-negative bacteria, including *Acinetobacter, Pseudomonas*, *Stenotrophomonas*, and *Enhydrobacter*. The effect of CO_2_ on Proteobacteria was completely opposite to Firmicutes (Figure 4A). This may be attributed to their competition and interaction (37), with Proteobacteria having poor adaptability to CO_2_. Bacteroidetes, a Gram-negative bacterium of which *Chryseobacterium* is the main genus, also showed poor adaptation to CO_2_ in this study. The results of microbial succession footprint analysis indicated that the levels of *Acinetobacter* and *Pseudomonas* in CO_2_-treated milk were lower compared to the untreated group on any day until the milk spoiled. The proportion of *Enhydrobacter* or *Chryseobacterium* was significantly reduced (*p* < 0.01) in the carbonated raw milk compared to the untreated milk during the first 4 days, but they were spoiled at roughly similar proportions (Supplementary Table S2). *Acinetobacter, Pseudomonas*, and *Chryseobacterium* are common psychrophilic spoilage bacteria in raw milk, which can produce heat-resistant hydrolytic enzymes that disrupt the sensory quality of milk and dairy products (38–40). *Pseudomonas* was reported to be the dominant psychrophilic bacterium in spoilage raw milk, followed by *Acinetobacter* (39, 41). However, in our study, the relative abundance of *Acinetobacter* was consistently greater than *Pseudomonas*, until the sample spoilage on day 6, and the relative abundance of *Acinetobacter* was twice that of *Pseudomonas*. This may be attributed to the lower initial relative abundance of *Pseudomonas* in raw milk, which is closely related to the milking environment, animal health, and hygiene conditions for handling (42, 43). Interestingly, it was observed that the growth rate of *Pseudomonas* was much higher than that of *Acinetobacter* (Figure 5B), confirming that *Pseudomonas* has the fastest reproduction rate among psychrophilic bacteria in raw milk (39). When lower concentrations of CO_2_ were added to the raw milk, the inhibitory effect on *Pseudomonas* was not significant (Figure 4B). When the concentration of CO_2_ exceeded 1,000 ppm, the relative abundance of *Pseudomonas* significantly decreased and remained at a low level (less than 0.6%), showing a downward trend during the refrigeration period. These results were consistent with many previous reports (11, 14, 15). This may be because the aerobic properties of *Pseudomonas* make it unable to survive in low oxygen environments (44). *Pseudomonas* is widely recognized for its detrimental effects, the presence of its heat-resistant hydrolytic enzymes can lead to undesirable outcomes, such as sedimentation, condensation, bitterness and stratification in dairy products, thereby reducing their shelf life and resulting in significant economic repercussions (45). Moreover, the formation of a biofilm may augment the stability of these heat-resistant hydrolases, exacerbating the economic losses (3). Additionally, the high prevalence of antibiotic resistance genes in *Pseudomonas* may increase the risk of transmission to humans, thereby posing a significant public health concern (46). *Acinetobacter* was very sensitive to CO_2_, and CO_2_ at 500 ppm significantly inhibited its growth (Supplementary Table S2). Through footprint analysis of the sample treated with 2,000 ppm CO_2_, it was observed that, similar to *Pseudomonas*, the relative abundance of *Acinetobacter* also tended to decrease and remained at a relatively low level. Compared with the untreated spoilage bacteria, the relative abundance of *Acinetobacter* was 10 times lower at day 6 and 18 times lower at day 16, respectively. A previous study also reported the inhibitory effect of CO_2_ on *Acinetobacter* in raw milk (15). Despite Acinetobacter’s inferior protein hydrolysis activity compared to Pseudomonas, it exhibited a more robust fat hydrolysis activity, thereby expediting the liberation of free fatty acids, contributing to the emergence of lipid soluble flavor defects in milk and dairy products, including sour rot, pungent or soapy taste (47). Meanwhile, it may potentially diminish the foaming characteristics of milk. Therefore, reducing the proliferation of psychrophilic bacteria, such as *Pseudomonas* and *Acinetobacter*, during the refrigeration of raw milk by dissolving CO_2_ is of great significance for saving milk resources and improving the quality of dairy products.

Many studies have found that CO_2_ had a selective effect on *Serratia* (14, 21, 48), but this genus was not found in our study. This may be due to the absence of *Serratia* in the initial microbial communities of the raw milk, indicating that CO_2_ may have a more significant selective or inhibitory effect on existing bacterial genera. Therefore, the initial microbial community of raw milk is crucial for bacterial succession (14).

According to Loss and Hotchkiss (10), CO_2_ treatment of raw milk can raise the quality of dairy products. This investigation presented the effects of CO_2_ on the protein, fat, lactose, acidity and flavor of raw milk. Protein and lipid hydrolysis was found to be decreased by CO_2_ (Table 1). In raw milk, Ma et al. (13) discovered that a drop in pH lowered the activity of endogenous proteases, such as plasmin, but did not affect the activity of endogenous lipases. It was hypothesized that CO_2_ decreased protein hydrolysis by decreasing the activity of natural proteases in raw milk and decreasing the synthesis of proteolytic enzymes by microbes. However, limiting lipid hydrolase synthesis by microbes is the sole way to reduce fat hydrolysis (13). This was consistent with the significant effect of CO_2_ on protein over fat in our study. The reduction of lactose content by CO_2_ may be related to the selective effect of CO_2_ on LAB, which could decompose lactose into glucose and galactose (49). As the refrigeration time increases, the relative abundance of LAB under the action of CO_2_ increased, resulting in a stronger decomposition effect on lactose. Correlation analysis also found a strong negative correlation between lactose and *Leuconostoc* (Figure 7B). CO_2_ can be easily removed prior to processing by simple vacuum or agitation and gentle heating (10), which further enhances its suitability for use in raw milk. In order to avoid a direct decrease in acidity caused by the addition of CO_2_, we degassed it before measuring acidity. Although a large number of LAB were selected by CO_2_, the total number of bacteria was decreased, and the degassing treatment also removed natural CO_2_ in the raw milk. Besides, there was no difference in the organic acid content of milk, with the exception of lactic acid (8), so that the acidity increased more slowly over time in the CO_2_-treated group (Table 1). This was consistent with a previous study (8).

Flavor is the most crucial indicator for evaluating food quality, and milk is no exception. Therefore, we conducted volatiles and taste properties analysis in the control and CO_2_-treated (2,000 ppm) samples through E-nose and E-tongue, respectively. The results showed that the dissolution of CO_2_ significantly reduced the contents of volatile substances, such as aromatic compounds and organic sulfides, alcohols, aldehydes, ketones, hydrogen sulfides, methane and hydrocarbons (Figure 8A), increased the umami, richness, bitterness (milk flavor) and sweetness, meanwhile reduced the sourness of raw milk (Figure 8C). It was reported that fresh raw milk had fewer volatile compounds and that the type and amount increased with refrigeration time (24). This is consistent with the results of this study. Volatiles in raw milk are related to the decomposition of its own components and the metabolic activity of spoilage bacteria. For example, *Pseudomonas*, *Acinetobacter*, *Serratia* and others can produce proteases and lipases, leading to the production of sulfides and other odors in milk. Meanwhile, protein and fat in raw milk were decomposed under the action of endogenous enzymes and bacterial metabolic enzymes to produce aldehydes, ketones, alcohols and sulfides (50). Following the application of CO_2_ treatment, a notable decrease in the TBC in raw milk was observed (Figure 1A), particularly in the levels of *Pseudomonas* and *Acinetobacter* (Figure 4B). Consequently, this phenomenon resulted in a notable decline in the synthesis of proteases and lipases by spoilage bacteria, as well as a reduction in the resultant byproducts generated through the degradation of proteins and fats (Table 1). Collectively, these factors contribute to impeding the escalation of volatile content. The umami in food is imparted by glutamic acid and has been confirmed in breast milk (51). Glutamic acid was the most abundant free amino acid in raw milk, and its content attended to decrease with refrigeration time (52). This may explain the decreasing trend of umami over time in this study. A previous study showed that LAB can synthesize glutamic acid, and glucose can improve its production capacity (53). The biomarker in CO_2_-treated raw milk was LAB (including *Lactococcus*, *Lactobacillus*, and *Leuconostoc*), which can promote the breakdown of lactose to produce glucose and galactose. This has created a favorable condition for the increase in glutamic acid (i.e., umami) and sweetness in CO_2_-treated samples. The higher the fat content in raw milk, the stronger the “milk flavor.” And the increase in free fatty acids produced by fat hydrolysis corresponds to an increase in the likelihood of raw milk rancidity. CO_2_ treatment can increase the fat content in raw milk and reduce the free fatty acids produced by fat hydrolysis (Table 2). These may contribute to the “milk flavor” of CO_2_-treated samples and the sourness of control samples.

## 5 Conclusion

Our results demonstrated that CO_2_ can extend the cold storage time of raw milk in a dose-dependent manner. Gram-positive bacteria were selectively affected by CO_2_, while Gram-negative bacteria were inhibited. The examination of the microbial community succession’s footprint showed that CO_2_ changed the richness and structure of the microbial community throughout the entire process of cold storage, eventually replacing the main genus of spoilage bacteria from *Acinetobacter* and *Pseudomonas* with LAB. Additionally, CO_2_ decreased the rate of acidity increase, protein and fat breakdown, while somewhat accelerating the lactose breakdown. The flavor analysis showed that CO_2_ can reduce the production of volatile substances, increase the umami, richness, milk flavor, sweetness, and reduce the sourness of raw milk. The study provides new theoretical insights into the application of CO_2_ in raw milk.

## Data availability statement

The original contributions presented in the study are publicly available. This data can be found here: NCBI database with accession number PRJNA1016304 (https://www.ncbi.nlm.nih.gov/bioproject/PRJNA1016304).

## Author contributions

AZ: Formal analysis, Investigation, Methodology, Writing – original draft. JL: Formal analysis, Methodology, Writing – review & editing. MW: Investigation, Writing – original draft. NB: Writing – review & editing. DL: Project administration, Supervision, Visualization, Writing – review & editing. CW: Financial acquisition, Software, Validation, Writing – review & editing.
