# Supplementary material for: Footprint analysis of CO2 in microbial community succession of raw milk and assessment of its quality
Source: Front Nutr. 2023 Dec 19;10:1285653. doi: 10.3389/fnut.2023.1285653 (PMC10773745; doi:10.3389/fnut.2023.1285653)
Supplement: Supplementary file 1 [file Table_1.DOCX]

Supplementary Material

Table S1. Relative abundance of *Firmicutes*, *Proteobacteria*, *Bacteroidetes* and *Actinobacteria* among groups. Variations are considered significant (**P* < 0.05 and ***P* < 0.01) when target phlum from a given concentration was different from 0 ppm.

|  | 0 ppm | 500 ppm | 1000 ppm | 2000 ppm |
| --- | --- | --- | --- | --- |
| *Firmicutes* | 0.3499±0.054 | 0.5077±0.065** | 0.5464±0.067** | 0.6820±0.078** |
| *Proteobacteria* | 0.5965±0.078 | 0.4486±0.054** | 0.3960±0.080** | 0.2747±0.081** |
| *Bacteroidetes* | 0.0396±0.008 | 0.0292±0.008* | 0.0325±0.009 | 0.0211±0.007** |
| *Actinobacteria* | 0.0124±0.004 | 0.0135±0.006 | 0.0236±0.005** | 0.0206±0.005** |

Table S2. Relative abundance of different genera among groups. Variations are considered significant (**P* < 0.05 and ***P* < 0.01) when target genus from a given concentration was different from 0 ppm.

|  | 0 ppm | 500 ppm | 1000 ppm | 2000 ppm |
| --- | --- | --- | --- | --- |
| *Leuconostoc* | 0.0987±0.032 | 0.2161±0.085** | 0.2864±0.053** | 0.3639±0.021** |
| *Stenotrophomonas* | 0.2790±0.064 | 0.2490±0.057 | 0.2785±0.074 | 0.2457±0.024 |
| *Lactococcus* | 0.1796±0.043 | 0.1994±0.028 | 0.2014±0.014 | 0.2063±0.029* |
| *Acinetobacter* | 0.2001±0.051 | 0.1184±0.036** | 0.0692±0.025** | 0.0341±0.014** |
| *Lactobacillus* | 0.0571±0.012 | 0.0622±0.011 | 0.0671±0.012 | 0.0626±0.016 |
| *Pseudomonas* | 0.0682±0.015 | 0.0637±0.017 | 0.0296±0.012** | 0.0054±0.003** |
| *Chryseobacterium* | 0.0356±0.009 | 0.0318±0.007 | 0.0321±0.005 | 0.0222±0.001** |
| *Enhydrobacter* | 0.0112±0.002 | 0.0066±0.001** | 0.0062±0.002** | 0.0032±0.000** |
| *Actinomyces* | 0.0024±0.000 | 0.0027±0.001 | 0.0053±0.002** | 0.0063±0.001** |

Table S3. Relative abundance of *Stenotrophomonas, Acinetobacter, Lactococcus, Leuconostoc, Pseudomonas, Lactobacillus, Chryseobacterium* and *Enhydrobacter* among days. Variations are considered significant (**P* < 0.05 and ***P* < 0.01) when target genus population from a given day was different from the same day of 0 ppm group. Except, day 16 of 2000 ppm was different from day 6 d of 0 ppm.

|  | 2 d | | 4 d | | 6 d | | 16 d |
| --- | --- | --- | --- | --- | --- | --- | --- |
|  | 0 ppm | 2000 ppm | 0 ppm | 2000 ppm | 0 ppm | 2000 ppm | 2000 ppm |
| *Stenotrophomonas* | 0.4410±0.073 | 0.4137±0.081 | 0.2780±0.098 | 0.2784±0.071 | 0.0256±0.005 | 0.3194±0.083** | 0.1048±0.018** |
| *Acinetobacter* | 0.1198±0.032 | 0.0518±0.009** | 0.2329±0.049 | 0.0308±0.009** | 0.3656±0.982 | 0.0354±0.008** | 0.0206±0.005** |
| *Lactococcus* | 0.2274±0.033 | 0.2989±0.053* | 0.1743±0.038 | 0.2915±0.056** | 0.0458±0.011 | 0.2558±0.067** | 0.1073±0.015** |
| *Leuconostoc* | 0.0339±0.010 | 0.0190±0.003** | 0.1170±0.025 | 0.2364±0.051** | 0.2329±0.057 | 0.2182±0.057 | 0.6712±0.193** |
| *Pseudomonas* | 0.0089±0.002 | 0.0051±0.001 | 0.0715±0.021 | 0.0052±0.001** | 0.1855±0.052 | 0.0042±0.001** | 0.0033±0.001** |
| *Lactobacillus* | 0.0562±0.008 | 0.0990±0.028** | 0.0374±0.008 | 0.0716±0.023** | 0.0039±0.001 | 0.0688±0.018** | 0.0367±0.011** |
| *Chryseobacterium* | 0.0457±0.006 | 0.0390±0.009 | 0.0358±0.007 | 0.0197±0.006* | 0.0069±0.002 | 0.0231±0.005** | 0.0064±0.002 |
| *Enhydrobacter* | 0.0195±0.003 | 0.0050±0.001 | 0.0153±0.002 | 0.0039±0.001** | 0.0015±0.000 | 0.0029±0.001 | 0.0015±0.000 |
